# Therapeutic potential of virgin coconut oil in mitigating sodium benzoate- model of male infertility: Role of Nrf2/Hmox-1/NF-kB signaling pathway

**DOI:** 10.22038/IJBMS.2024.71288.15484

**Published:** 2024

**Authors:** Ayodeji Johnson Ajibare, Olabode Oluwadare Akintoye, Moshood Abiola Folawiyo, Kabirat Temitope Babalola, Olaposi Idowu Omotuyi, Busayo Timothy Oladun, Kafilat Temidayo Aransi-ola, Adeyemi Fatai Odetayo, Luqman Aribidesi Olayaki

**Affiliations:** 1 Department of Physiology, Faculty of Basic Medical Sciences, College of Medicine and Health Sciences, Lead City University Ibadan, Oyo State, Nigeria; 2 Department of Physiology, Ekiti-State University, Ado-Ekiti, Ekiti-State, Nigeria; 3 Department of Pharmacology& Toxicology, College of Pharmacy, Afe Babalola University, Ado-Ekiti, Ekiti-State, Nigeria; 4 Federal University of Health Sciences, Ila Orangun, Osun State, Nigeria; 5 Department of Physiology, Faculty of Basic Medical Sciences, College of Health Sciences, University of Ilorin. Nigeria

**Keywords:** Food additives, Reproductive hormones, Sperm analyses, Testicular toxicity, Virgin coconut oil

## Abstract

**Objective(s):**

Male infertility is a major public health issue due to increased prevalence, so there is an urgent need for a therapeutic solution. The search for a natural dietary substance that could modulate redox balance and inflammation and protect testicular function is in demand. Virgin Coconut Oil (VCO) has found use in the treatment of diabetes, and cancer owing to the presence of polyphenols. However, there is a dearth of information on its effect on testicular toxicity. The present study investigated VCO as a possible treatment for testicular toxicity in the Sodium Benzoate (SB) model of male infertility by evaluating the oxidative and inflammatory status, circulating hormonal levels, and key sperm indices.

**Materials and Methods:**

Twenty adult male rats were randomly assigned to four groups of 5 rats each and were treated with normal saline, sodium benzoate, sodium benzoate+5% VCO, and sodium benzoate+15% VCO for 30 days respectively. Biochemical analysis of reproductive hormones was assessed. Sperm parameters assessed include sperm function tests and sperm kinematics. One-way analysis of variance (ANOVA) followed by post hoc Tukey tests was performed.

**Results:**

5% VCO reverts the deranged serum reproductive hormones caused by sodium benzoate. 5% VCO was more potent as an antioxidant and anti-inflammatory treatment than 15% VCO. However, both doses prevented SB’s effect on the sperm function test and kinematics.

**Conclusion:**

VCO-supplemented diet can ameliorate SB-induced testicular toxicity by inhibiting its mechanisms of toxicity that are related to oxidative stress, apoptosis, and inflammation.

## Introduction

Globally, infertility affects more than 70 million individuals, with the male population affected in approximately half of the total cases. Infertility in men is a complicated condition that is typically caused by multiple factors, and it has its roots in genetics, immunological or endocrine pathologies (1), biotic (urogenital infections), and abiotic (endocrine disruptors)(2) contributors ultimately resulting in failed testicular development and spermatogenic dysfunction (3). Clinically, male infertility resulting from testicular failure is seen as unmodifiable, while assisted reproductive techniques (ART) have benefited some male patients with compromised spermatogenesis (4). 

Undoubtedly, a broadened understanding of the underlying pathological mechanisms of male infertility will be vital to developing clinically actionable therapeutic agents or other treatment options (5). 

Sodium benzoate (SB) is a well-known food additive that is an emerging endocrine disruptor. In a previous study, adult male Wistar rats given oral SB showed altered testicular cytoarchitecture, resulting in lowered sperm quality, disrupted endocrine function of the testis, and increased oxidative stress status without change in body mass (6). In a similar study, 90 days of orally administered SB dose-dependently increased the percentage of abnormal sperms, altered the weight of reproductive organs, and decreased sperm parameters (7). Interestingly, the changes were associated with testicular inflammatory, apoptotic, and oxidative changes, which are classical phenotypes of failed testicular development and spermatogenic dysfunction. 

Clomiphene treatment is a bold attempt at reversing Leydig and Sertoli cellular secretory dysfunction associated with male infertility (8). The underlying mechanism is increased circulating LH, FSH, and testosterone, resulting in improved sperm count/quality, morphology, and motility (9). In this study, however, virgin coconut oil (VCO) was investigated. 

VCO is derived from fresh coconut (*Cocos nucifera*), which has found medicinal use in the treatment of diabetes, diarrhea, and cancer (10), owing to the presence of polyphenols, tocopherols, sterols, and squalene (11). These phytocompounds have been reported to improve semen quality (quantity, quality, and motility)(12) but not in SB-induced testicular toxicity. 

The current study, therefore, investigated VCO as a possible treatment for testicular toxicity in the SB model of male infertility by evaluating the oxidative and inflammatory status, circulating hormonal levels and their cognate receptors, and key sperm kinematic indices. 

## Materials and Methods

***Chemicals***

May and Baker Ltd. Dagenham, England supplied the sodium benzoate salt. The Sigma Chemical Company, St. Louis, Missouri, USA, provided the other chemicals.

***Animals***

Twenty mature male Wistar rats weighing between 160 and 180 g were donated by the Ekiti State University’s animal house in Ado-Ekiti, Ekiti-State, Nigeria. The rats were kept in plastic cages with adequate ventilation at Ekiti State University’s Animal Laboratory in Ado-Ekiti, Ekiti-State, Nigeria, under regular lighting, feeding, and temperature conditions (13). The rats had full access to rat food and water for the duration of the experiment. The protocol this study) EKSU/P100/2021/11/010 (was approved by the Ekiti State University College of Medicine’s ethics and research committee. 

***Experimental protocol***

The experimental protocol was designed such that after an acclimatization period of 14 days, the animals were randomly assigned (minimum n=5 per group) to the following treatments:

Control: Administered 1 ml normal saline solution via oral gavage

NaB: Sodium benzoate monotherapy at 200 mg/kg body weight via oral gavage (14)

NaB+Low VCO: Sodium benzoate plus 5% virgin coconut oil incorporated into 95 g standard chow

NaB+High VCO: Sodium benzoate plus 15% virgin coconut oil incorporated into 85 g standard chow

***Preparation of supplemented chow containing virgin coconut oil***

This supplemented chow containing VCO was processed according to Azubuike-Osu *et al*. (15). Matured and fresh coconuts were bought in a local market in Ado-Ekiti, Nigeria. Grated coconuts and tepid water were blended and strained into a thick paste to extract all creamy milk. This creamy milk was fermented for 48 hr at room temperature. The layers were VCO, water, and a creamy concoction. Filtered VCO was collected. The supplemented diet was created by weighing 5% and 15% w/w VCO from the fresh VCO and adding them to 95 g and 85 g of conventional rat feed, respectively, and mixed well (16).

***Preparation of serum for biochemical assay***

On the last day, the animals were fasted all night. On day 29, xylazine/ketamine (10/50 mg/kg)(17) was administered intraperitoneally to induce anesthesia, and animals were subsequently sacrificed for the biochemical analysis of serum GnRH, FSH, LH, and GnRH using the manufacturer’s kits; blood was drawn through a retro-orbital sinus puncture, put into non-heparinized tubes, and spun at 3000 rpm for 15 min.

***Gene expression***

Following the manufacturer’s instructions, the first of each set of testes was cut in half longitudinally. This allowed for the expression of genes. Total RNA isolation was carried out using the TRIzol reagent (ThermoFisher Scientific), followed by DNAse I treatment to remove all traces of DNA from the isolated RNA (ThermoFisher Scientific), followed by conversion of the RNA into cDNA using the ProtoScript® First Strand cDNA Synthesis Kit (NEB), and finally amplification by polymerase chain reaction using forward and reverse primer sets and OneTaq® 2X Master mix (NEB). Table 1 shows all the genes that were analyzed.

***Sperm analysis***

The computer-assisted sperm analyzer determined sperm parameters according to Akhigbe and Ajayi (18) with some modifications. Briefly, the left caudal epididymis of each testis was dissected. Several incisions of about 1 mm were made on it to liberate its spermatozoa before placing it in a clean petri dish containing 2 ml of normal saline solution. Sperm parameters include sperm function tests, sperm kinematics, and Sperm velocities.

***Preparation of tissue for histology***

The second part of the left testis was fixed in sufficient amount of bouin’s fluid and routinely prepared and embedded with paraffin. Five micrometer slices were cut and stained with hematoxylin and eosin (H&E) for histology evaluation.

***Statistical analysis***

Graph-pad Prism version 9 was used to compare the groups (*P*<0.05) using one-way ANOVA and *post hoc* Tukey testing.

## Results

The HPG axis strictly regulates the generation of testosterone in male reproduction. Higher cortical areas boost hypothalamus’ production of GnRH. To govern the generation of testosterone by Leydig cells and spermatogenesis by Sertoli cells, GnRH stimulates the anterior pituitary to release the gonadotropins LH and FSH. The synthesis of GnRH and the gonadotrophins decreases when testosterone levels rise due to negative feedback inhibition on androgen receptors in pituitary and hypothalamus neurons (19). The “short loop” process of negative feedback reduces male prolactin levels (20). Estradiol and testosterone affect libido, erectile function, and spermatogenesis in men. Independently, low testosterone and high estrogen induce erectile dysfunction (21).

**Figure 1:**
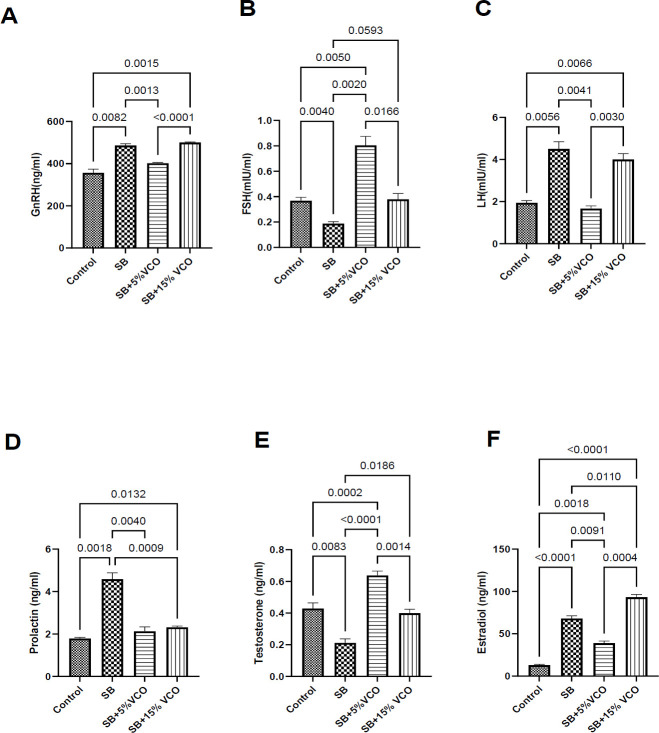
Low dose VCO reverts the deranged serum reproductive hormonal profile of SB-induced male rat

Figure 1 shows that the serum concentrations of GnRH of the control group are significantly lower compared to the remaining groups (*P*=<0.0001, <0.0001, <0.0001, respectively), and our 5% VCO treatment dose ameliorates this derangement better as it showed a significantly lower serum concentration of GnRH compared to the negative control and 15%VCO treated animals (*P*=<0.0001, <0.0001, respectively). The serum FSH of the low-dose treated rats was higher than that of the control and high-dose VCO-treated rats (*P*=<0.0001, 0.045, respectively), correcting the defect seen in the SB animals. Serum LH of the SB group was significantly lower when compared to the other groups (*P*=<0.0001, <0.0001, <0.0001, respectively). Both low and high doses corrected this derangement (*P*=<0.0001, <0.0001) in serum prolactin. Our results also showed that low-dose VCO reverses the significantly low serum level of testosterone and high level of estrogen seen in the SB groups compared to the high dosage (*P*=0.012, <0.0001, respectively). 

Numerous system disorders, including reproductive abnormalities, which can lead to issues with spermatozoa production and infertility, have oxidative stress as a factor in their origin. By breaking down hydrogen peroxide, an antioxidant by the name of catalase neutralizes it, preserving an ideal concentration of the molecule in the cell, which is required for cellular signaling (22). The activity of glutathione reductase (GSR) is a sign of oxidative stress. Reduced glutathione, the most prevalent reducing thiol in most cells, is kept in check by GSR. In its reduced state, glutathione regulates reactive oxygen species in cells (23). Nrf2 regulates several antioxidant responses and element-dependent genes’ basal and induced expression to control oxidant exposure’s physiological and pathological impacts (24). The oxidative stress sensor HMOX1 has catalytic metabolites with antioxidant, anti-apoptotic, and anti-inflammatory activities, such as CO and biliverdin (25). Oxidative stress accelerates the apoptosis process. The primary effector caspase activated by initiator caspases during the execution of apoptosis is caspase-3. Once activated, the protease cleaves the building blocks of the cytoskeleton and DNA, resulting in irreversible self-destruction that defends the cells (26).

**Figure 2:**
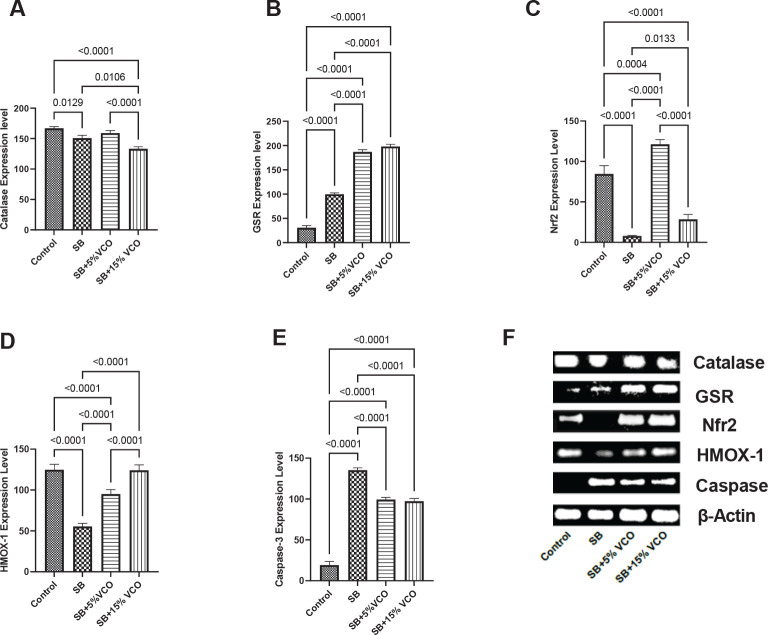
A-E: Low VCO exhibits better anti-oxidative properties than high-dose VCO in SB-induced testicular toxicity in rats

This anti-oxidative function is more evident in Figure 2a. Both catalase and GSR are potent endogenous antioxidants. Our study showed that 5% VCO significantly up-regulates the expression of catalase in the testicular homogenate of SB-induced testicular toxicity when compared to high dose treatment group (*P*<0.0001) but non-significantly higher compared to the SB rats (*P*=0.416). This involves both the Nrf-2 and HMOX-1 pathways, as demonstrated in Figures 2c and 2d, evident in the two dosages compared to the SB animals. Similarly, both 5% and 15%VCO treated animals showed the moderate anti-apoptotic feature statically as it repressed caspase-3 mRNA expression compared to SB rats (*P*<0.0001 and <0.0001, respectively)(Figure 2e).

**Figure 3:**
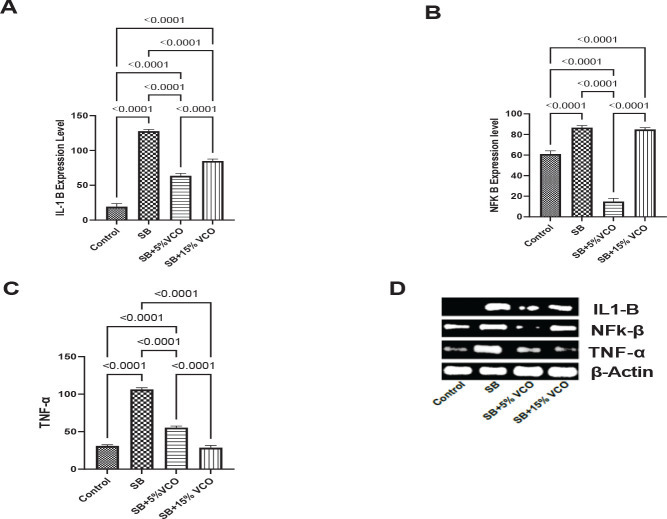
-C. Low-dose virgin coconut oil displays greater anti-inflammatory effects than high-dose in sodium benzoate-induced male infertility model

Inflammation is mediated by NF- κB. NF- κB stimulates the production of pro-inflammatory genes like cytokines and chemokines and regulates inflammasomes (27). Cytokines play a key role in inflammation. Specifically, higher levels of the cytokines interleukin-1 beta (IL-1β) and tumor necrosis factor-alpha (TNF-α) in the blood have been associated with more robust and escalating immune system responses to infections and diseases (28). According to our findings in Figure 3a, inflammation in SB was significantly more than in the control group, whereas IL-1B levels in the 5% and 15% VCO-treated groups of rats were significantly lower (*P*=<0.0001) than those in the SB and 15% VCO-treated groups. The figure shows the up-regulated gene expression of pro-inflammatory cytokine TNF-α in SB-treated rats. 3b was also significantly reversed by 5% and 15% VCO (*P*=<0.0001). However, 15% VCO was significantly lower when compared to 5% VCO (*P*=<0.0001). The level of NFK-B mRNA expression, as seen in Figure 3c, was significantly higher in SB-treated animals compared to the control group. However, it was significantly reversed only in the 5% VCO group (*P*=<0.0001). 

A network of the hypothalamus, pituitary, and gonads controls the production of gametes and steroid hormones. Gonadotropin-releasing hormone, at the center of the network, stimulates the production and secretion of LH and FSH by binding to receptors on pituitary gonadotrophs after it is released from the brain. Once they enter the circulation, LH and FSH connect to their receptors and send instructions to the gonads to finish the communication (29). Follicle-stimulating hormone receptor (FSHR) is found on the ovary granulosa cells and the testis’ Sertoli cells. As a result, the expression of FSHR regulates the targets and the level of FSH activity, finally directing the hormone response to Sertoli cells in the testis and granulosa cells in the ovary (30). Luteinizing hormone receptors can be seen on the theca and granulosa cell surfaces. In response to luteinizing hormone binding to it, hormones such as androstenedione, progesterone, and testosterone are produced (31).

**Figure 4:**
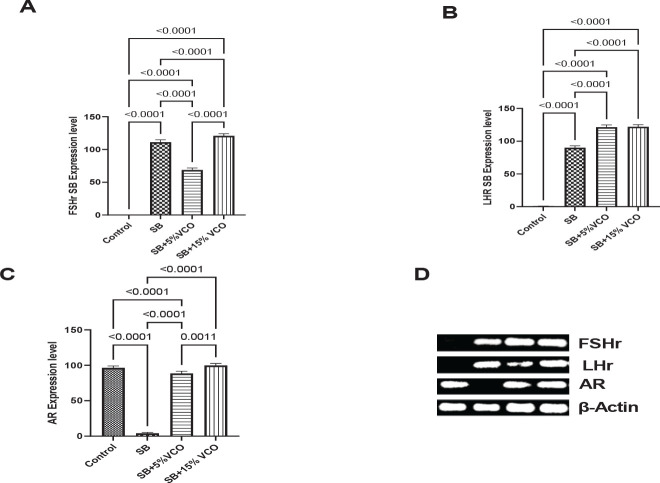
Increased fertility potential of 5% VCO in sodium benzoate model of male infertility

Contrarily, androgen receptors (AR) interact with other proteins in the nucleus to change the transcription of a certain gene. The creation of messenger RNA is then translated into specific proteins by ribosomes and is increased when transcription is activated or up-regulated. Therefore, during different stages of ontogenesis, AR mediates the masculinizing effects of androgens on distinct reproductive system components (32). Figure 4a shows that 5% VCO could down-regulate the FSH receptor significantly compared to the SB group (*P*=<0.0001); however, 15% VCO could not reverse it. Figure 4b reveals that the androgen receptor was down-regulated in the SB group compared to all other groups (*P*=<0.0001, <0.0001, <0.0001, respectively). However, 5% VCO up-regulated the androgen receptor better than the 15% VCO group (*P*=0.001).

 The sperm must be adequate in terms of count and typical structure (morphology). Sperm need to have a membrane that is both intact and able to function properly in order for them to be able to endure the harsh environment of the vaginal and uterine fluids (vitality). Sperm also need to have a good mitochondrial function for them to be able to provide energy (motility) in addition to their other functions (33).

Table 2 shows that the sperm count of the SB group is significantly higher than both the control and treatment groups (*P*=<0.0001, <0.0001, respectively). The morphology index of the SB group was significantly lower than both control groups and treatment groups (*P*=<0.0001, <0.0001, respectively). However, the 5% VCO group ameliorated the morphological defects better than 15% VCO (*P*=0.0052). The 5% VCO group restored sperm vitality with a significant increase compared to 15% VCO (*P*=0.002). However, the sperm vitality of the SB group was significantly lower than both control groups and treatment groups (*P*=<0.0001, <0.0001, respectively). The total sperm motility of the SB group was significantly lower than both the control and treatment groups (*P*=<0.0001, <0.0001, respectively). However, 5% VCO restored it better than 15% VCO (*P*=0.001). Fast progression motility was significantly lower in the SB group compared to control and treatment groups (*P*=<0.0001, <0.0001, respectively), this was reversed in 5% VCO when compared to SB and 15% VCO (*P*=<0.0001, <0.0001, respectively). Slow progression motility was significantly lower than control, SB, and 15% VCO (*P*=<0.0001, <0.0001, <0.0001, respectively). The 15% VCO group had a significantly better non-progression than the 5% VCO group (*P*=0.005). At the same time, the SB group had significantly lower non-progression motility than both the control and treatment groups (*P*=<0.0001, <0.0001, respectively). The SB group had a significantly higher level of immotile sperm than both the control, 5% VCO, and 15% VCO groups (*P*=<0.0001, <0.0001, <0.0001, respectively); however, the 5% VCO group had a significantly reduced level of immotile sperm compared to 15% VCO (*P*=0.029). 

A crucial aspect of sperm motility that can provide insights into the sperm’s ability to penetrate the zona pellucida is the assessment of frequencies related to smooth and non-smooth motion patterns. Notably, when considering curvilinear velocity (VCL) as a metric averaged over a trajectory, it may fail to surpass the required threshold for classifying a trajectory as hyperactivated if there is a transition in sperm movement patterns from hyperactive to non-hyperactive within the analyzed timeframe (34). The smoothing-dependent kinematics variables provided by computer-assisted sperm analysis (CASA) include average path velocity (VAP), which is the length of the derived ‘average’ path/time, straightness (STR), wobble (WOB), and amplitude of lateral head displacement (ALH)(35).

In contrast to the control and treatment groups, the SB group’s average path velocity (VAP) is significantly lower than both of them (Table 3; *P*=<0.0001 and *P*<0.0001, respectively), although it was significantly improved by 5% VCO (*P*<0.0001). The 5% VCO group achieved a superior straight-line velocity (VSL) restoration compared to the 15% VCO (*P*=0.0026). However, the VSL of the SB group was considerably lower than that of the control, 5% VCO, and 15% VCO groups (*P*=<0.0001, *P*=0.002, and *P*=<0.0001, respectively). 

The Curvilinear Velocity (VAP) of the SB group was significantly restored by 5% VCO compared to SB and 15% VCO groups but was significantly lower in the control and treatment groups (*P*=<0.0001, *P*<0.0001 and *P*=0.0032, respectively). The amplitude of lateral head displacement (ALH) was considerably lower in the 5% VCO group compared to the control, SB, and 15% VCO groups (*P*=<0.0001, <0.0001, and <0.0001, respectively), whereas 15% VCO was statistically higher than other groups (*P*=<0.0001, <0.0001, and <0.0001, respectively). Beat cross frequency (BCF) of the SB group is significantly lower than that of the control and treatment groups (*P*=<0.0001, <0.0001, respectively) but was significantly restored more effectively by 15% VCO when compared to SB and 5% VCO groups (*P*=<0.0001, <0.0214, respectively). Both the 5% and 15% VCO groups’ linearity, straightness, and wobbling were statistically higher than the SB group’s (*P*=<0.0001, <0.0001, respectively), while the SB group’s was statistically lower than the control group’s (*P*=<0.0001). Both the 5% and 15% VCO groups’ Straightness scores were statistically higher than the SB group’s (*P*=0.0138 and *P*=0.0005, respectively), but the SB group’s value was statistically lower than the control group’s (*P*=0.0147). Compared to the control group, the SB group’s wobbling significantly decreased (*P*=0.0007). Statistically, the 5% and 15% VCO groups significantly outperformed the SB group (*P*=0.078 and *P*=0.0005, respectively). 

**Figure 5:**
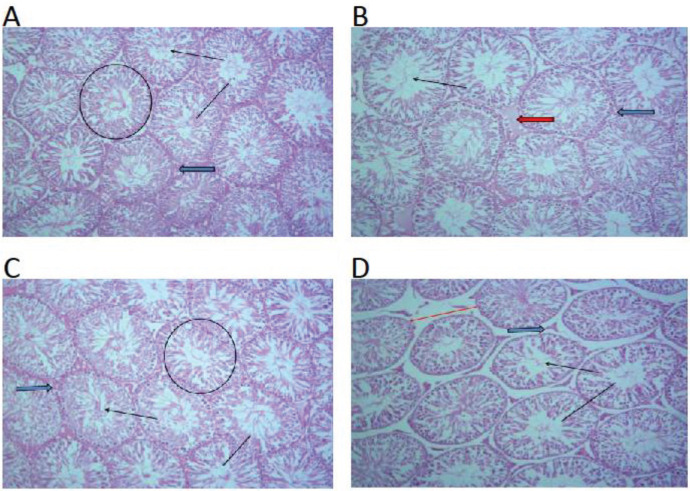
Virgin coconut oil protected the integrity of the testes in the model of male infertility

Figure 5 shows photomicrographs of the testicular tissue (H&E stains) X 100 of Control, SB, and treatment groups (A-D).

**Table 1:** List of genes analysed and their corresponding primers

GENE	FORWARD PRIMER	REVERSE PRIMER
NRF2	GTCAGCTACTCCCAGGTTGC	CAGGGCAAGCGACTGAAATG
HMOX-1	CGACAGCATGTCCCAGGATT	AGGAGGCCATCACCAGCTTA
CATALASE	TGCTCCCAACTACTACCCCA	AGAATGTCCGCACCTGAGTG
GLUTATHIONE REDUCTASE	CTTCTCACCCCAGTTGCGAT	GTGGCTGAAGACCACGGTAG
NFKB	CCACTGTCAACAGCAGATGG	TTCTTCTCACTGGAGGCACC
IL1B	CAGCTTTCGACAGTGAGGAGA	TGTCGAGATGCTGCTGTGAG
TNF A	ATGGGCTCCCTCTCATCAGT	GCTTGGTGGTTTGCTACGAC
ANDROGEN RECEPTOR	AAAAGAGCTGCGGAAGGGAA	TTTCCGGAGACGACACGATG
FSH RECEPTOR	ATTCTTGGGCACGGGATCTG	TGGTGAGCACAAACCTCAGTT
CASPASE 3	GAGCTTGGAACGCGAAGAAA	GAGTCCATCGACTTGCTTCCA

The bar charts indicate the standard error of each sample’s mean (n = 5) gene/actin ratio of the 1.5% agarose in the TAE buffer image. They were calculated using the ImageJ computer program. The photographs capture the sample as a whole. One-way ANOVA was done using Graph-pad Prism version 9 with a significance threshold of 0.05 for statistical analysis

The data is presented as the mean±the standard error of the mean (SEM). The sample size (n) for each group is 6. A one-way analysis of variance (ANOVA) statistical test was conducted, followed by a Tukey *post hoc* test to compare the differences between the means of the groups. Where GnRH, FSH, LH, SB, and VCO are gonadotropin-releasing hormone, follicle-stimulating hormone, luteinizing hormone, sodium benzoate, and virgin coconut oil, respectively

Demonstrates the testicular antioxidant gene expression patterns in the Control, SB, SB+5%VCO, and SB+15%VCO (A–D) groups. The mean (n = 6) across the four groups represents the values of the measured bands from each sample for a particular gene. A one-way ANOVA was used to assess the data, followed by Tukey’s multiple *post hoc *test. The gel picture is an accurate representation of the combined samples. Each bar graph displays a particular gene’s normalized relative control expression of a particular gene or -actin. SB, VCO, NRF2, HMOX-1, and CASP-3 are abbreviations of Sodium Benzoate, Virgin Coconut Oil, Nuclear Factor-Erythroid-Related Factor 2, Heme Oxygenase 1, and Caspase 3, respectively

Demonstrates anti-inflammatory gene transcription profiles in the Control, SB, SB+5%VCO, and SB+15VCO groups. Each sample’s measured band values for a particular inflammatory gene are expressed as mean ±SEM (n = 6) over the course of the four groups. One-way ANOVA and Tukey’s multiple *post hoc* analysis were used to analyze the data. An accurate representation of the combined samples may be found in the 3D gel image. Each bar graph shows the relative control normalized expression of an individual gene or -actin. Where TNF –α, NFkb, and IL-1B represent Tumor Necrotic Factor- alpha, Nuclear Factor Kappa B, and Interleukin 1B, respectively

Demonstrates the patterns of testicular expression of hormone receptor (Fshr, LHr, and AR) genes in Control, SB, SB+5%VCO, and SB+15VCO. The values of the measured bands from each sample for a particular inflammatory gene are expressed as mean±SEM (n=6) across the four groups. A one-way ANOVA followed by Tukey’s multiple post hoc test was used to examine the data. The image of the gel is a representative snapshot of the combined samples. (each bar graph depicts the relative control normalized expression of a certain gene or -actin). SB (Sodium Benzoate); VCO (Virgin coconut oil); AR (Androgen receptor), FSHr (Follicle-stimulating hormone receptor)

**Table 2:** Virgin coconut oil mitigates deranged sperm function tests in SB-model of male infertility

	Control	SB	SB+5%VCO	SB+15%VCO
Sperm count (million/ml)	28±1.16^b^	37.85±1.02	18.35±0.72^b^	14.85±0.64^b^
Morphology index (%)	54.67±1.35	30.67±1.33^a^	51.17±1.68^b^	43.17±1.49^ab^
Vitality (%)	98.72±0.43^b^	62.7±1.5	99±0.51^b^	92.33±1.56^b^
Total motility	72.83±1.4^b^	17.65±1.71	69.52±1.76	59.15±1.79^abc^
Fast progression motility	45.29±1.41^b^	0.38±0.13	43.32±1.0^b^	24.97±0.97^abc^
Slow progression motility	2.37±0.14^b^	2.06±0.21	0.09±0.06^b^	2.04±0.08^ab^
Non progression motility	24.52±1.08	15.1±0.87	26.2±0.60	31.5±1.26
Immotile	27.63±0.76	71.75±3.42	30.7±1.35	39.88±1.99

Data are presented as the mean±standard error of the mean (SEM), and each group consists of n=6 samples. Statistical analysis involved employing one-way analysis of variance (ANOVA), followed by Tukey’s *post hoc* test to assess group differences. Significance is denoted by distinct lowercase letters (a, b, c, d) at a P-value threshold of <0.05. Specifically, ‘a’ signifies significance in comparison to the control group, ‘b’ indicates significance relative to the sodium benzoate (SB) group, ‘c’ represents significance when compared to the SB group, and ‘d’ denotes significance in relation to the SB+5% virgin coconut oil (VCO) group

**Table 3:** Virgin coconut oil ameliorates deranged sperm kinematics in SB-model of male infertility

	Control	SB	SB+5%VCO	SB+15%VCO
VAP (μm/s)	12.63±0.44^b^	4.12±0.17	10.42±0.33^b^	7.81±0.29^b^
VSL (μm/s)	7.24±0.25	1.72±0.07^a^	5.99±0.18^b^	5.05±0.28^ab^
VCL (μm/s)	20.76±0.48^b^	9.09±0.24	18.09±0.44^b^	16.05±0.14^b^
ALH (μm)	1.19±0.01^b^	0.79±0.01	0.485±0.01	1.198±0.01^abc^
BCF(Hz)	1.87±0.01^b^	1.31±0.04	1.545±0.02^b^	1.735±0.06^abc^
Linearity	0.349±0.01^b^	0.191±0.01	0.33±0.01^b^	0.315±0.02^ab^
Straightness	0.57±0.03	0.425±0.03	0.579±0.02	0.646±0.03
Wobble	0.61±0.03	0.454±0.02	0.575±0.01	0.488±0.02

Data are presented as mean±standard error of the mean (SEM) with a sample size of n=6 per group. One-way analysis of variance (ANOVA) followed by Tukey’s *post hoc* test was used to analyze differences between groups. Groups denoted with different lowercase letters (a, b, c) are significantly different from each other at *P<*0.05. Specifically, ‘a’ indicates statistical significance compared to the control group, ‘b’ denotes significance versus the sodium benzoate (SB) group, and ‘c’ refers to significance compared to the SB+5% virgin coconut oil (VCO) group. Abbreviations are as follows: SB - sodium benzoate, VCO - virgin coconut oil, ALH - amplitude of lateral head displacement, BCF - beat cross frequency, VAP - average path velocity, VSL - straight line velocity, VCL - curvilinear velocity

## Discussion

The widespread use of the chemical food preservative sodium benzoate (SB) in pharmaceuticals and nutraceuticals has aroused significant concerns about its hidden harmful effects. An increasing amount of research demonstrates the negative impacts of SB on human health, particularly its adverse effects on reproduction (7). According to studies, SB increases redox imbalance, mitochondrial damage, and inflammation in the testes, leading to apoptosis (3, 7). Studies have demonstrated that VCO has biological qualities both *in vitro *and* in vivo*, including antioxidant and anti-inflammatory effects (36, 37). Our study aimed to ameliorate the detrimental effects of SB using dietary VCO. 

We found that SB deranged sperm function tests in rats in the present study. This correlates with previous studies which showed that SB impairs male reproductive health (38). We observed that SB significantly reduced the morphology index, mobility, progressivity, and vitality of sperm cells, indicating rats’ lack of fertilizing capacity due to their exposure to the toxicant SB. This is consistent with the findings of a previous study, which showed that exposure to SB reduces sperm motility and leads to aberrant sperm morphology. However, we noticed a significant increase in the amount of sperm in the group that had been treated with SB, which contradicts the findings of a prior study that claimed SB exposure lowered the number of sperm (6, 38).

Our research has revealed that exposure to SB has a detrimental effect on sperm motility in rats. Notably, rats subjected to SB treatment exhibited noteworthy decreases in various aspects of sperm movement, encompassing average path velocity, curvilinear velocity, linearity, wobble, straightness, straight linear velocity, lateral head displacement, and beat cross frequency. These findings strongly suggest that SB disrupts the typical patterns of sperm motion crucial for fertility. It is important to note that this represents the first evidence indicating the adverse influence of SB on sperm kinematics and, consequently, reproductive potential. In addition, the histological results revealed vascular congestion within the interstitial space. This correlates with previous studies that show that SB induces histological alterations in the testicular tissue of rats (38). However, consumption of the VCO diet mitigated the deranged sperm function tests, ameliorated deranged sperm kinematics, and prevented histological changes in testicular tissue in SB-treated rats.

Follicle-stimulating hormone (FSH) and luteinizing hormone (LH) both regulate gene expression and cell-to-cell communication during the process of spermatogenesis. Both hormones are released and controlled by the HPG axis, which does so in response to the hypothalamus via the gonadotropin-releasing hormone (GnRH). FSH is in charge of controlling the growth, development, and functionality of the Sertoli cells that support other cells. These cells provide the regulatory signals and essential nutrients to promote expanding germ cells, both independently and in combination with testosterone (39, 40). 

The results of this study corroborate with those of others by showing that SB treatment significantly raises plasma GnRH, LH, estrogen, and prolactin levels while simultaneously decreasing plasma FSH and testosterone (7, 41). This might be responsible for the derangement of the sperm function tests and sperm kinematics observed in the SB-treated group, as the deficiency of FSH stimulus in rodents has been documented to decrease sperm quantity significantly (39). However, consuming a VCO diet reversed these hormonal deviations. 

Increased expression of FSH-R is a potential molecular marker of testicular disorders (42). Evidence suggests that sodium benzoate can be chemically converted into the carcinogenic compound benzene (43). In this study, we observed up-regulation of the FSH receptor in the SB-treated group. The carcinogenic potential of SB might be responsible for the observed increase in the FSH receptors. This outcome is consistent with earlier research linking higher FSH-R expression to advanced testicular cancer stage (43). Additionally, research has shown that the deletion of the AR gene in mouse germ cells affects the maturation of the male phenotype but not spermatogenesis or male fertility. In the SB-treated group, we detected a down-regulation of the androgen receptor. The phenotype of the SB-treated rats is unaffected by this, though, as all of their organs were fully formed before exposure to SB. These anomalies were mitigated by VCO diet, indicating that VCO may have some anticancer potential, according to an earlier study (44).

Sodium benzoate’s toxicological effects on oxidative stress and inflammatory cytokines are well-established (3, 7, 45). Oxidative stress is characterized by insufficiency of antioxidants or radical scavengers relative to the amount of reactive oxygen species (ROS) generated (46). The ability of sodium benzoate to produce a carcinogenic metabolic product increases the possibility of generating reactive free radicals. An increase in redox imbalance caused by SB results in rapid consumption of the antioxidant components present, thus lowering their concentration in the body system (14). 

It was observed that SB caused a significant reduction in the serum level of the antioxidant SOD and down-regulated the expression of catalase, a potent endogenous antioxidant. This is consistent with previous research that found sodium benzoate to increase oxidative stress (14, 45). By raising catalase expression and SOD serum levels, the VCO diet reduced the oxidative damage generated by SB. This is consistent with previous studies describing the rejuvenating effect of VCO on antioxidant enzymes in the face of toxicity (16). The antioxidant effect of VCO can be attributed to its potent phenolic compounds. Also, the antioxidant ability of VCO, which is responsible for its ability to reduce oxidative stress, might be responsible for its ability to prevent testosterone suppression and testicular damage, which was observed in this study (47).  

The improved sperm morphology, vitality, and motility parameters observed after VCO supplementation indicate a protective effect on sperm maturation and function. SB likely induced oxidative damage and inflammation in the testicular microenvironment, disrupting spermatogenesis and resulting in abnormal, immotile sperm. The oxidative stress-reducing and anti-inflammatory properties of VCO likely preserved the testicular niche allowing for normal sperm development and the release of higher quality sperm. The normalized testosterone levels with VCO treatment could also contribute to the improved sperm parameters through androgen-mediated support of spermatogenesis (40). 

The transcription factor Nrf2 is essential for the prevention of oxidative stress-induced disruption of spermatogenesis. It achieves this by regulating the constitutive and inducible transcription of genes that code for enzymes involved in reducing oxidative stress (48). In addition, the heme oxygenase system, which consists of heme oxygenase proteins, protects cells against the detrimental effects of oxidative stress (49). In this investigation, we noticed a significant decrease in the Nrf2 and HMOX 1 gene expression in the SB-treated group. As studies have shown, a deficiency in the Nrf2 and HMOX 1 genes leads to testicular and epididymal oxidative stress, diminished antioxidant capacity, and loss of cytoprotection, all of which disrupt spermatogenesis (48, 50, 51).

Additionally, caspase-3 mRNA expression increased in the SB-treated group. This shows an increase in apoptosis in the SB-treated rats, which may indicate testicular cancer because studies indicate that the proliferation ratio to apoptosis may be higher in carcinomas (52). The consumption of a VCO‐supplemented diet up-regulated the expression of both Nrf2 and HMOX 1 gene and down-regulated the expression of the caspase-3 mRNA, correlating with previous research, which has shown that VCO has cytoprotective, anti-apoptotic, anticancer, and antioxidant properties (44, 53).

When the induced free radicals and antioxidant enzymes are out of balance, it leads to significant tissue damage and raises levels of important inflammatory cytokines (54). These cytokines are formed during the chronic phase of tissue injury and circulate systemically throughout the body (14). Strong evidence supports that oxidative stress enhances the production of nuclear factor kappa B (NFkB) (55). In numerous inflammatory cascades, the transcription factor NF-kB controls the production of cytokines. NFB is transported to the nucleus when it is active, where it promotes the expression of inflammatory genes. The mechanism of inflammation via NFB activation results in SB toxicity (14). In line with earlier research, we discovered an increase in NFB expression in rats given SB. This is consistent with past findings that SB causes significant tissue damage and activates the NFB signaling system, increasing the production of the cytokines TNF- and IL-1B, as seen in the current study (14, 17). The marked increase in the TNF-α and IL-1B can lead to inflammatory testicular damage (14). The consumption of a VCO-supplemented diet reversed all of the observed pro-inflammatory changes. The consumption of the VCO diet significantly down-regulated the expression of inflammatory genes such as NF-ĸB, IL-1β, and TNF-α. This is in tandem with prior studies that documented VCO’s ability to reduce pro-inflammatory responses (16). 

## Conclusion

Our findings suggest that the VCO-supplemented diet can ameliorate SB-induced testicular toxicity by modulating the Nrf2/HMOX 1 pathway and down-regulating the NF-κB/caspase signaling pathway. 

## Authors’ Contributions

A AJ and A OO conceived the project, A AJ, A OO, and F MA carried out the project, B KT, O BT, A KT, O AF worked on the initial draft. A AJ, A OO, and O AF performed the statistical analysis, and O IO and O LA supervised. All authors read and approved the final version of the manuscript.

## Funding information

This study had no funding or financial support.

## Conflicts of Interest

The authors declare no conflicts of interest.
